# Supplementary material for: Somatosensory Intervention Targeting Temporomandibular Disorders and Awake Bruxism Positively Impacts Subjective Tinnitus
Source: Audiol Res. 2025 Sep 9;15(5):114. doi: 10.3390/audiolres15050114 (PMC12452508; doi:10.3390/audiolres15050114)
Supplement: Supplementary file 1 [file audiolres-15-00114-s001.zip › audiolres-3763381-supplementary.pdf]

Supplementary material

Table 1S: Tinnitus Functional Index, Pre- and Post-Treatment per Participant

SN	TFI T0	TFI T1	Δ TFI	Δ TFI %
1	42	7	-35	83%
2	55	17	-38	69%
3	70	61	-9	13%
4	42	24	-18	43%
5	33	24	-9	27%
6	43	20	-23	53%
7	44	18	-26	59%
8	48	28	-20	42%
9	43	28	-15	35%
10	46	26	-20	43%
11	29	13	-16	55%
12	65	34	-31	48%
13	69	51	-18	26%
14	37	32	-5	14%
15	67	58	-9	13%
16	49	44	-5	10%
17	33	32	-1	3%
18	56	23	-33	59%
19	38	25	-13	34%
20	41	38	-3	7%
21	57	49	-8	14%
22	86	62	-24	28%
23	28	27	-1	4%
24	14	14	-0	0%
25	75	30	-45	60%
26	30	1	-29	97%
27	39	0	-39	100%
28	43	16	-27	63%

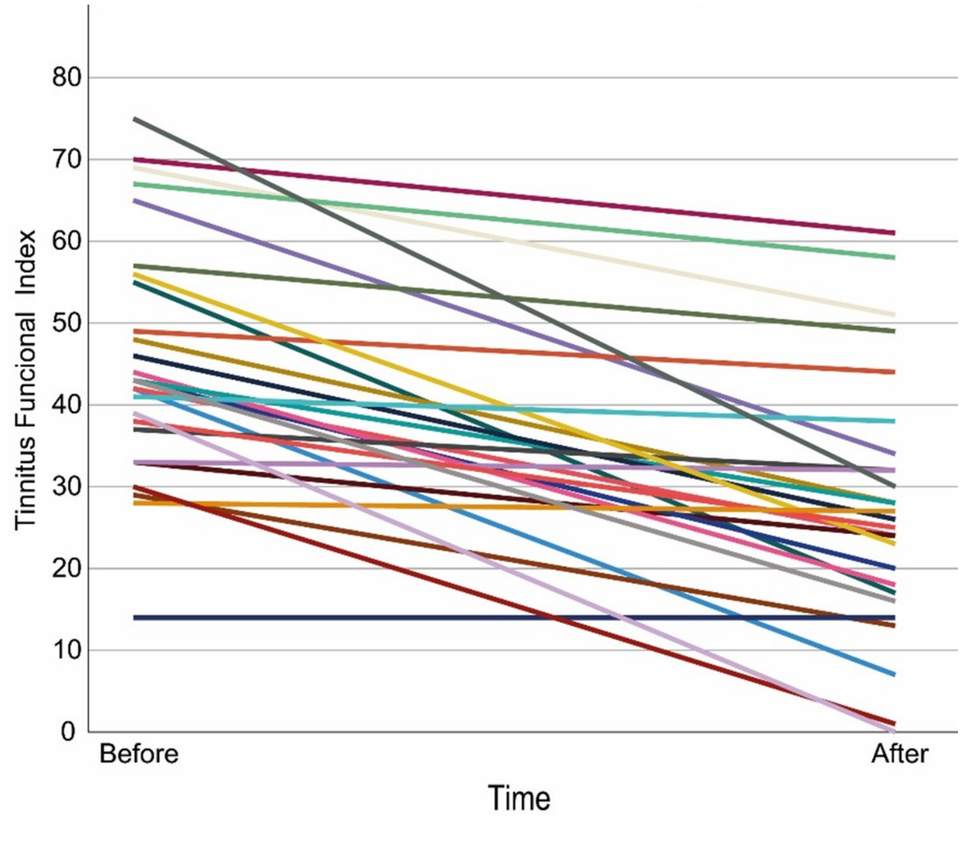

Figure 1 (color): Tinnitus Functional Index over time per Participant
